# Farewell Address of the President, Dr. Davis

**Published:** 1865-07

**Authors:** 


The President of the Association, Dr. Davis, then delivered his farewell address, which was as follows:—

Gentlemen:—I thought when I was down upon the island under that cottage roof, posted upon a chair, that the emotions within me were such as almost entirely to forbid utterance; but I confess, notwithstanding the warm congratulations and cordial feelings which surrounded us there, they have fallen below what you have put upon me to do at the close of this meeting, in the manifestations of your approval of the manner in which I have been permitted to discharge the duties of my office. Compelled last year by the amendment of the constitution to serve two years in these same labors, I find so warm a manifestation of your approval as surely will never be forgotten by me; this time has been an enjoyment that will leave its impress upon the deepest recesses of my heart while I am permitted to live, no matter what may be the vicissitudes that await me in the future, or changes which may take place in this our national organization. The feelings which have been impressed upon me here will remain unobliterated while time lasts, and I fondly hope will not be wasted while eternity itself shall endure. But, gentlemen, I must, in turn, offer my most cordial thanks to every one of you, for if we have had excellent meetings, it is because you, every one of you, have kindly sustained me in the discharge of my duty, and, therefore, I say, I am as much indebted to you, and far more than you are to me; therefore, I give you my thanks. And I tender my thanks cordially to the City of Boston, and to all those in the vicinity, for the manner in which I, as an humble individual, have been treated. Before I close, let me make one more observation. I have been very strongly impressed for twelve months past with the feeling that this Association had arrived at a crisis of its existence; and I have felt at the opening of the session, this meeting here in the old cradle of liberty, almost in sight of the waves of the Atlantic, would determine whether this Association was to be perpetuated to the latest generations, gathering the profession from the four quarters of the continent, not merely from East or West, or North or South, but from the whole continent, and uniting it in a common brotherhood for defending the interests of humanity, as long as civilization itself shall endure—or whether we should culminate, and, like the transient meteor in the heavens, make our mark and fade away. And I felt strongly impressed at the time, because I saw many aged among us, and many young who looked to the aged, and because they did not come up to their expectations fully, were hanging back, and inquiring why? And it seemed we had arrived exactly at the period where the Association was about to be transferred from one generation to the next, and as I commenced very young, I felt that I stood as a connecting link, that my right hand was on the shoulder of the aged, and my left upon the shoulder of the younger members of the profession, and if I could hold them together and put the burden of the Association over from the shoulders of the one fairly upon the other, I felt that it would stand firm through the coming trial. My brethren of the profession, I feel that the crisis has passed. [Cheers.] The Association is established upon the firm shoulders of the next generation. I am sure they will pass it to the next and along to others as long as civilization shall exist, therefore I cordially sympathize with you in all the enjoyments of this occasion; and I hope, while I am permitted to live and be able to go by steam-ship or rail-car, that I will find time to enjoy a green oasis every year, in meeting you as long as age shall let me linger with you. [Cheers and applause.]

At the close of the address of Dr. Davis, three hearty cheers were given for that gentleman.

At a quarter past one o'clock, the Convention adjourned to meet again on the first Tuesday in May, 1866, in the City of Baltimore.

The annual meeting of the Association of 1865 has been more largely attended than on any previous occasion, six hundred and sixteen members and delegates having registered their names on the books of the committee. It is generally regarded as having been a most interesting and successful convention.

*A Visit to the Readville Hospital.*—After the adjournment, a considerable number of the physicians availed themselves of an invitation which had been received to visit the U.S.A. General Hospital, at Readville, free passes having been tendered by the Providence Railroad Company. On the arrival of the party at Readville, they were welcomed by the surgeon in charge, who, with his assistants, afforded the visitors every facility for examining the institution. The officers of the Hospital are now as follows:—Dr. Gross, Surgeon of Volunteers, in charge, and Acting-Assistant-Surgeons Wilbur, Langmaid, Osborne, and Ropes.

